# Toward sustainability in zeolite manufacturing: An industry perspective

**DOI:** 10.3389/fchem.2022.1050363

**Published:** 2022-12-06

**Authors:** Andrei-Nicolae Parvulescu, Stefan Maurer

**Affiliations:** ^1^ BASF SE, Ludwigshafen, Germany; ^2^ BASF Catalysts Germany GmbH, Hannover, Germany

**Keywords:** zeolite, industry sustainability, manufacturing, catalysis, chemical industry

## Abstract

Conventional zeolite manufacturing processes are highly energy-intensive and come along with a significant carbon dioxide footprint. Here, we discuss the main energy consumers and potential alternatives toward a more sustainable production of zeolites: from simple optimization efforts on existing unit operations to new and novel manufacturing concepts such as the continuous crystallization and solidothermal route toward zeolites and their industrial applicability. These efforts contribute to the global effort into transitioning manufacturing of chemicals and catalysts to a net-zero environment by cutting greenhouse gas emissions to as close to zero as possible.

## Introduction

**SCHEME 1:**
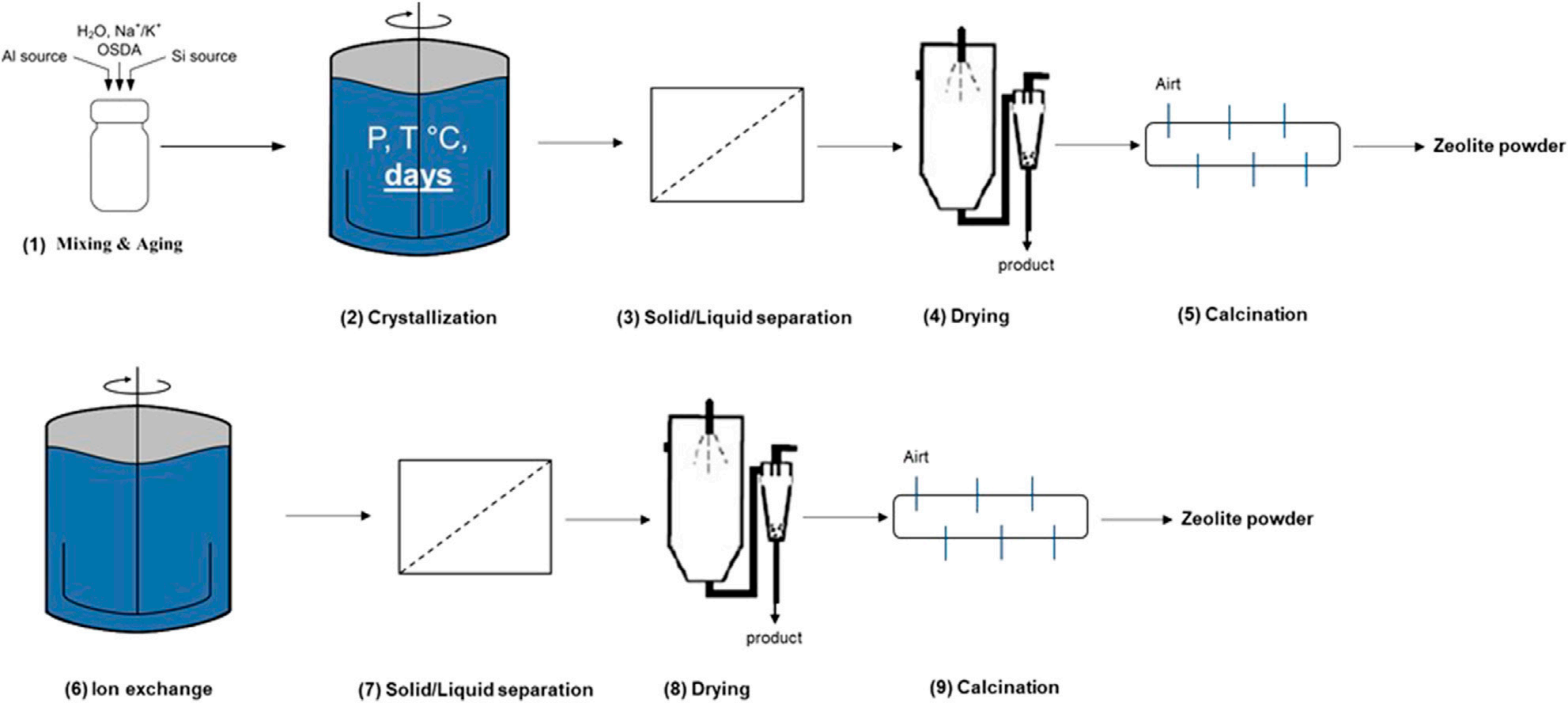
General zeolite manufacturing scheme.

Zeolites continue being one of the most relevant classes of catalysts for both chemical and petrochemical industries. Their intrinsic properties in terms of variable framework geometries, tunable compositions, and accessible pore structure of molecular dimensions enabled their use as catalysts or adsorbents in many industrial relevant processes (Yilmaz and Müller, 2009), and they are expected to further play a crucial role in a future sustainable industry. Conventional production of industrially relevant zeolite frameworks changed little over the last 70 years and involves multiple manufacturing steps, several of them operated in a batch mode and others continuously. As a classical example, the synthesis of an aluminosilicate zeolite would be starting with (1) gel preparation by mixing silica + alumina, an organic structure-directing agent (OSDA), and inorganic mineralizers. The resulting gel is subjected to the (2) crystallization step by applying hydrothermal conditions in a pressurized autoclave. Depending on the particle size of the resulting zeolite crystals, an optional agglomeration is required before the zeolite is subdued to (3) solid/liquid separation and washing off the excess of the OSDA and other byproducts. To burn off the organic template occupying the pore structure of the zeolite framework, the resulting filter cake first needs to undergo (4) drying followed by (5) calcination at elevated temperatures. The furbished zeolite powder often needs further downstream processing steps: the active catalyst is obtained by one or two subsequent (5) ion exchanges, followed by another (6) solid/liquid separation plus the washing step and successive (7) drying and optionally (8) calcination (Scheme 1). For most industrial applications in catalysis, the resulting zeolite powder needs to be further subdued to a shaping or coating process to furbish the finished form of the catalyst. The aforementioned process is generally applied to non-aluminosilicates as well, although some of the processing steps could be skipped, such as, for example, ion-exchanges.

Zeolite manufacturing in general is a highly energy-intensive process, with the steps 2 + 4 + 5: crystallization, drying, and calcination, respectively, as the major energy consumers due to the need for elevated temperatures over extended periods of time for these particular unit operations. Even though zeolite-based catalysts during their lifetime significantly contribute to more efficient chemical processes, in turn reducing the overall CO_2_ footprint of large industrially relevant applications and playing an important role in environmental catalysis reducing harmful NO_x_ emissions from diesel cars and trucks and stationary sources; there is a strong incentive toward a more sustainable zeolite manufacturing process.

Driven mostly by academic research, around 246 of the zeolite framework topologies were synthesized, not including the intergrowth structures. These materials could be produced in various compositions, and additionally over 20,000 structures are known, hypothetically. Despite this, less than 15–20 zeolite structures made their way into industrial applications. In addition to the potential lack of applications, the transfer of zeolite recipes from an early-stage academic environment into an industrial setup is not trivial. Some of the aspects that are mostly overlooked in academia, but are industrially relevant, are as follows:

• Space time yield of the recipe: mostly expressed as kilogram product, per volume of the synthesis reactor and time. This gives an indication of the synthesis yield, with lower values indicating long synthesis time (high energy consumption) or lower reactant yields (high volumes of wastewater).
• Raw material selection: some syntheses involve expensive and complicated organic structure-directing agents, which would require complex synthesis procedures with high amounts of waste/high energy consumption. Sometimes, mineralizing agents such as fluorides or other inorganics are used which present issues in terms of EHS and off-gas/wastewater handling. Raw materials could be an important source of the CO_2_ footprint in zeolite manufacturing; although, they would not be a direct emitter in the process itself.
• Energy of synthesis gels is often an overlooked aspect in academia: this refers to the stability of the raw materials during hydrothermal synthesis and the risk of thermal/catalytic decomposition, resulting in increased pressure or runaway reactions.

In this paper, we address concepts that could improve the sustainability directed toward zeolite manufacturing steps such as synthesis, drying, and calcination.

## Zeolite manufacturing steps: Gel preparation and crystallization

The conventional crystallization process involves heating up the synthesis gel to elevated temperatures (120–200°C) over an extended period of time (20–100 h) to allow the crystallization of the desired zeolite framework. The heating of the autoclaves typically occurs indirectly *via* a secondary heating loop powered by steam or natural gas firing, which in turn can be translated into the generation of CO_2_. The CO_2_ footprint per kilogram of zeolite depends strongly on the gel composition and the conduct of crystallization; quite often well above 1 kg CO_2_/kg of the zeolite product. These constraints are nicely captured within the concept of *space-time-yield*, which describes the amount of zeolite produced in a given crystallization time per volume of the corresponding reactor. The higher the solid content in the gel, the shorter the crystallization time, and the higher the *space-time-yield*. As the amount of energy required to heat up and maintain the crystallization at the target temperature is more or less independent from the gel composition, a higher *space-time-yield* not only means a higher efficiency in the utilization of the given equipment but also a huge lever to reduce the energy consumption per kilogram of the zeolite.

**FIGURE 1:**
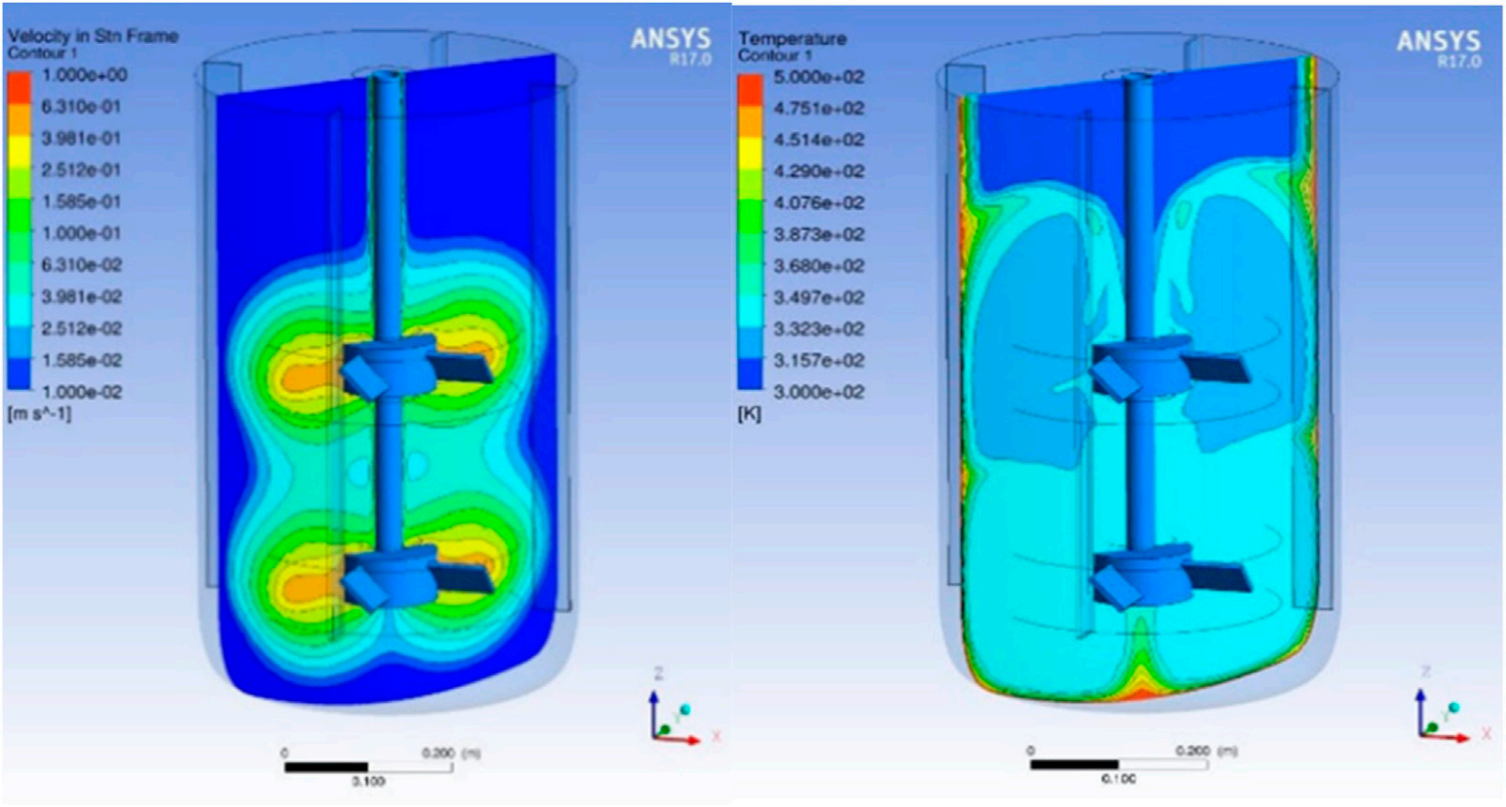
CFD analysis of a zeolite synthesis gel in a stirrer autoclave during zeolite crystallization.

Typically, zeolite recipes prone to industrial production undergo a series of optimization steps to reduce the overall crystallization time. The first measure is the control of the crystallization progress, *ex situ* by taking samples for the determination of the degree of crystallization and *in situ* by implementing the speed of sound sensors to follow the progression of the particle size distribution (Toufar, 2010). Commonly employed strategies to increase the speed of crystallization comprise modification of the gel composition, in particular the ratio of the template/mineralizer to SiO_2_, adding an aging period at temperatures below < 100°C prior to crystallization not requiring an autoclave (Hikichia et al., 2019), addition of seeds (Mochida et al., 1997), or simply an increase in crystallization temperatures (Bian et al., 2018). Sticking to the conventional hydrothermal batch process, however, has limitations in the maximum achievable crystallization speed even for highly optimized crystallization recipes. The sheer thermal mass of the reactor + synthesis gel *vs.* the available heating/cooling capacity for a typical commercial autoclave (10–20 m^3^) comes with heat-up and cool-down times of at least a couple of hours each, while the minimum residence time for crystallization amounts from hours to days. The thermal lag results in heterogeneities in terms of heat distribution within the synthesis reactors, which could influence the nucleation and crystal growth process of zeolite synthesis (Liu et al., 2014a). Furthermore, a CFD analysis (see Figure 1) showed that even for well-stirred and heated reactor gradients, mixing flows and temperature distributions could occur during the hydrothermal treatment. Microwave heating can speed up the heat-up and crystallization to a faster pace (Zeng et al., 2021); realization on a larger scale is, however, extremely challenging, and still cooling down the reactor to temperatures below 100°C is required.

Hence, new concepts for the crystallization process to overcome these constraints are of high relevance not only for academia. Moving away from the conventional batch concept to continuous crystallization, using a tubular flow reactor is one promising path toward ultra-fast crystallization (Thome et al., 1992; Liu et al., 2019). Due to the favorable surface-to-volume ratio, a tubular reactor allows bringing the synthesis gel to temperatures well above 100°C within less than a minute. By applying a careful gel preparation strategy to overcome kinetic barriers in nucleation by addition of seeds and an aging period, Liu et al. (2014b) demonstrated the crystallization of AlPO_4_ within minutes. The synthesis concept could soon be extended to several industrial relevant zeolites, including SSZ-13 (Liu et al., 2015) and TS-1 (Schreyer et al., 2021). The pre-activation of the synthesis gel can also be achieved *via* mechanochemical activation (Parvulescu et al., 2020). Such a mechanochemical activation route involves only a fraction of the time of conventional aging.

In parallel to reducing the residence time in a reactor, increasing the solid content of a given synthesis gel results in higher *space-time-yields* and a lower overall energy consumption per kilogram of the zeolite product. In addition, the continuous flow approach could be used for zeolite post-treatment as recently exemplified by Wakihara et al. (2020) through mesopore formation into zeolites.

Taking the solid content in a given crystallization process to its extreme—the removal of all water from the synthesis gel—was demonstrated first back in the early 90s in the so-called dry gel conversion (Xu et al., 1990), respectively, dry gel synthesis (Althoff et al., 1994), followed by the introduction of the solidothermal synthesis concept by Xiao et al. (Wu et al., 2015) in 2015. The latter approach introduced ammonium fluoride as a mineralizer, which is thoroughly mixed with the other anhydrous raw materials *via* intense grinding, enabling a completely solvent and steam-free crystallization process, also unlike earlier approaches, where no solvent-based precursor synthesis is required. Omission of water as a solvent allows applying higher temperatures exceeding 200°C, which under conventional conditions require high-pressure resistance of the employed autoclaves and further contribute to a more rapid degradation of the OSDA *via* the Hoffmann elimination and other decomposition pathways. As crystallization speed goes hand in hand with the applied temperature, the solidothermal approach allows a faster overall crystallization at high solid content. The same synthesis strategy can also be applied to zeolite phase transformations; high silica CHA could be obtained by interconverting high silica FAU in the absence of water as a solvent, adding seeds and OSDAs as a bromide salt, furbishing a zeolite with a very comparable NO_x_ conversion to a conventionally prepared CHA (Xiong et al., 2017). Operating at high solid concentrations has the overall advantage of producing much less wastewater, in turn reducing the environmental footprint of the synthesis step.

Another highly interesting concept to reduce overall emissions is the organic structure directing agent (OSDA)-free synthesis. OSDAs mostly consisting of quaternary ammonium or phosphonium salts are generally applied in the synthesis of high Si or specialty zeolites. Although highly effective toward the synthesis of target zeolite structures and compositions, these organic molecules are often costly and require special handling (EHS issues). Since they are mostly burned during the zeolite calcination, special off-gas treatment is required to handle the COx, NOx, and other emissions. In this sense, minimizing the amount of such OSDAs in the synthesis gel and applying simple and less toxic molecules are preferred for industrial synthesis (Meng and Xiao, 2014; Wu et al., 2019). A completely OSDA-free synthesis would be an even more preferred alternative.

New synthesis routes to frameworks that generally require the use of an OSDA, such as BEA and high silica CHA SSZ-13, were reported without the need of an OSDA (Xiao et al., 2009; Kamimura et al., 2010). Using a seed-assisted OSDA-free synthesis, Xiao and his co-workers succeeded in synthesizing a BEA zeolite, with the composition and physical properties found different to the ones obtained through the known OSDA synthesis routes (Xie et al., 2011; Yilmaz et al., 2013). This result was successfully extended to other frameworks of interest, such as CHA, LEV, and FER, while the recent results showed that this approach can be applied as a general valid synthesis concept (Itabashi et al., 2012). Through this method, the highly energy-intensive calcination step could be removed, while the TOC content in the wastewater is significantly reduced as well. More importantly for the applications, new materials showing new properties, for example, in terms of acid site concentration and strength, could open up new possibilities of applications in catalysts or as adsorbents, e.g., in CO_2_ storage and gas-purification.

The synthesis mechanism behind the zeolite OSDA-free synthesis was studied by different research groups. Particularly, the role and proper selection of the seed crystal and the composition of the synthesis gel were found critical for the success of this synthesis route (Iyoki et al., 2013). The presence of Na and its concentration in the synthesis gel was found to be important as well since it acts as an inorganic directing agent in zeolite synthesis. Xiao and his coworkers demonstrated that the OSDA-free synthesis concept could also be successfully applied in the solidothermal synthesis of zeolites (Wu et al., 2014). The combination of both the approaches allows the synthesis of industrially relevant frameworks such as BEA or MFI at high *space-time-yields* under the avoidance of emissions to both wastewater and air.

**FIGURE 2:**
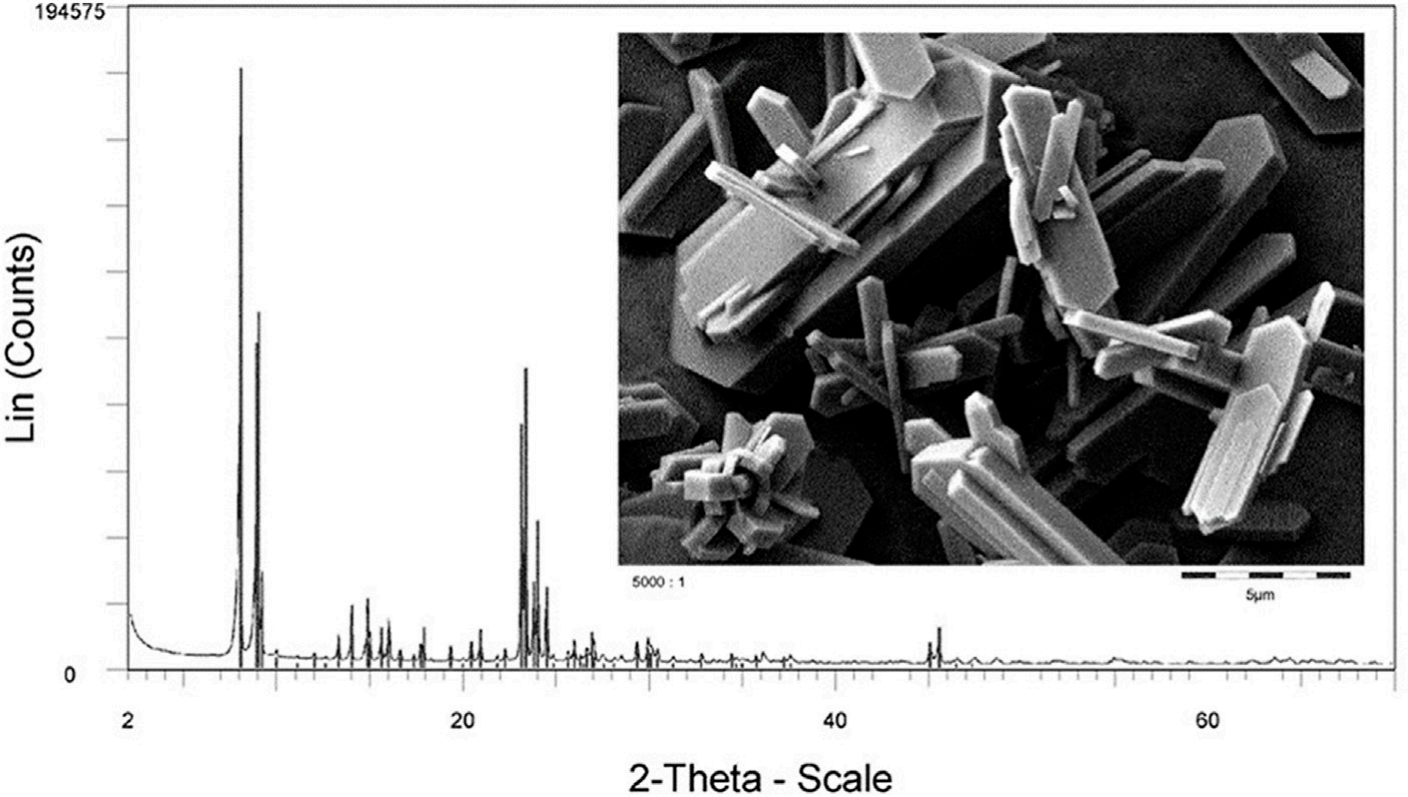
Silicalite from elemental silicon.

Use of waste as a raw material for zeolite synthesis is another approach to increase the sustainability of zeolite crystallization. Quite often untransformed raw materials, OSDAs, or Si/Al species, for example, are separated and recycled into the synthesis gels, rather than being disposed. In addition, waste streams from other chemical manufacturing processes could be used as raw materials for zeolite synthesis. An example in this sense is the use of silicon waste coming from the production of solar panels. This source of Si was shown to be used in the synthesis of high-value MFI zeolites, such as TS-1 (Maurer et al., 2015) (Figure 2).

Moving toward sustainability and net-zero in the industrial manufacturing of zeolites requires researchers in academia and the industry to consider energy consumption and generation of emissions already during the very early research phase when developing new materials or recipes. Proper selection of the crystallization method and the respective equipment and raw materials and the corresponding structure directing agents is critical on this path.

## Zeolite manufacturing steps: Drying and calcination

In addition to crystallization, the drying step, typically conducted in spin flash or spray dryers (Gleichmann et al., 2017), and the calcination process employing rotary kilns or belt calciners (Ulisch et al., 1990; Wu and Cao, 2020) are the main energy consumers in the overall zeolite manufacturing progress. In the drying process, the wet zeolite particles are dried by moving through hot air, with the air typically heated up to the required temperatures (300–500°C) by natural gas firing. A direct measure to reduce the overall energy consumption is using heat content of the hot process air coming out of the dryer to pre-heat the incoming air. Modifying the solid content in the feed slurry along with a window of the drying temperature and/or the targeted hourly throughput can further contribute to optimizing the overall energy consumption in the drying step. The heating of the process air can also be converted to electrical heating—switching to green electricity, in turn, allows an emission-free operation of the zeolite drying step, although with the overall industry trend moving toward electrification, competition for green electricity is expected to get more and more intense.

Typical temperatures applied during calcination are in the range between 500 and 650°C under oxidizing conditions to burn off the organic structure inside the zeolite framework (Choudhary and Singh, 1985). Heat recovery by using the hot process gas to pre-heat the incoming air to reduce the need for firing the kiln is possible as well. Likewise, belt calciners and rotary kilns can be converted from natural gas-fired toward electrically heated calciners, in turn switching to green electricity. The development of the OSDA-free zeolite synthesis renders the calcination step completely obsolete, a very attractive route also due to the avoidance of complex exhaust after-treatment systems to remove HC, NO_x_, and CO emissions and the overall simplification of the post-treatment process. Furthermore, as the calcination process puts thermal stress on the integrity of the framework, potentially leading to dealumination or even partial collapse of the porous framework, the avoidance of such a step can also improve the overall quality of the zeolite powder and in turn furbish a catalyst with enhanced catalytic activity, e.g., *via* higher preservation of active sites in the framework.

Another interesting approach for the case, the addition of OSDAs to the synthesis gel cannot be circumvented, is the merging of the drying and calcination steps into one single process step. This is achievable by applying the concept of flash calcination (Kalo et al., 2017). The wet zeolite suspension is atomized to form an aerosol and a dried version and calcined at the same time. Due to the way shorter residence time being in the milliseconds range in a flash calciner compared to a conventional calcination (rotary kiln 0.5–2 h; belt calciner up to 6 h), way higher temperatures can be applied (exceeding 1,000°C). This allows effective removal of the OSDA despite the extremely short retention time ranging from 40 to 160 ms. The short residence time also allows an overall much better temperature control than in conventional calciners, where local hot spots can easily lead to partial damage of the zeolite framework. The energy need for higher temperatures in the flash calciner is overcompensated by the combination of two steps into one.

The new learnings in terms of drying and calcination of zeolites have the potential to apply less energetically intensive processes with less thermal stress on the zeolite framework.

## Conclusion

There exist multiple pathways toward a more sustainable zeolite production, reducing the overall environmental footprint for this industrially highly relevant class of materials. Electrification allows the simple conversion of existing unit operations without major changes to the already present zeolite production processes. Moving toward the OSDA-free zeolite synthesis can likewise make use of the existing production infrastructure, while fully eliminating the need for a highly energy-intensive calcination step and also dramatically decreasing wastewater generation and other emissions. Applying new concepts such as the continuous zeolite synthesis in a tube reactor or the solidothermal zeolite crystallization does face a higher barrier toward the realization being put into steel and iron. The intensifying push toward carbon dioxide neutrality by both the society and customers is, however, a strong driving force not only for academia but also for the relevant zeolite manufacturers to explore alternative and novel concepts to produce zeolites that during their lifetime not only act as catalysts contributing to the reduction of the environmental impact but also result from a low and green energy consumption-based production process.

## Data Availability

Dedicated to Dr. Ulrich Müller on the occasion of his 65th birthday.

## Data availability statement

The data analyzed in this study are subject to the following licenses/restrictions: these datasets belong to BASF SE; part of the data **is** published (cited in the references) in forms of academic publications or patents. Requests to access these datasets should be directed to andrei-nicolae.parvulescu@basf.com.
